# A novel classification method for balance differences in elite versus expert athletes based on composite multiscale complexity index and ranking forests

**DOI:** 10.1371/journal.pone.0315454

**Published:** 2025-01-30

**Authors:** Yuqi Cheng, Dawei Wu, Ying Wu, Youcai Guo, Xinze Cui, Pengquan Zhang, Jie Gao, Yanming Fu, Xin Wang

**Affiliations:** 1 School of Exercise and Health, Shenyang Sport University, Shenyang, China; 2 School of Exercise and Health, Shanghai University of Sport, Shanghai, China; 3 School of Winter Olympic, Harbin Sport University, Harbin, Heilongjiang, China; 4 Sports Diagnosis and Evaluation Professional Technology Innovation Center of Liaoning, Shenyang, China; Ningbo University, CHINA

## Abstract

Balance is crucial for various athletic tasks, and accurately assessing balance ability among elite athletes using simple and accessible measurement methods is a significant challenge in sports science. A common approach to balance assessment involves recording center of pressure (CoP) displacements using force platforms, with various indicators proposed to distinguish subtle balance differences. However, these indicators have not reached a consensus, and it remains unclear whether these analyses alone can fully explain the complex interactions of postural control. In this study, we investigated four parameters related to balance control—anterior-posterior (AP) displacement, medial-lateral (ML) displacement, length, and tilt angle—in 13 elite athletes and 12 freestyle skiing aerial expert athletes. Data were recorded during 30-second trials on both soft and hard support surfaces, with eyes open and closed. We calculated the CMCI and used four machine learning algorithms—Logistic Regression, Support Vector Machine(SVM), Naive Bayes, and Ranking Forest—to combine these features and assess each participant’s balance ability. A classic train-test split method was applied, and the performance of different classifiers was evaluated using Receiver Operating Characteristic(ROC) analysis. The ROC results showed that traditional time-domain features were insufficient for accurately distinguishing athletes’ balance abilities, whereas CMCI performed the best overall. Among all classifiers, the combination of CMCI and Ranking Forest yielded the best performance, with a sensitivity of 0.95 and specificity of 0.35. This nonlinear, multidimensional approach appears to be highly suitable for assessing the complexity of postural control.

## 1 Introduction

Balance is a critical component of athletic performance, influencing an athlete’s ability to execute movements with precision and stability. In sports, optimal balance can be the difference between success and failure, particularly in disciplines that demand high levels of coordination and control [1,2]. Winter sports, for example, rely on a delicate equilibrium to achieve optimal performance and avoid potential mishaps [3]. Freestyle aerial skiing elevates the importance of balance to a whole new level, as athletes execute intricate aerial maneuvers requiring extraordinary coordination and control. A recent freestyle aerial skiing event at the Beijing Winter Olympics highlighted this challenge, revealing a substantial 32.56% failure rate in landings, underscoring the pivotal role of successful landings in determining competition outcomes [4].

Balance refers to the process of maintaining the center of gravity (CoG) vertically over the base of support, which relies on rapid and continuous feedback from the visual, vestibular, and proprioceptive systems [5]. This integration facilitates the efficient execution of neuromuscular actions, making balance a critical factor for athletes striving for outstanding competitive performance. Superior balance has been shown to correlate significantly with performance indicators across various sports, with athletes at higher competitive levels exhibiting stronger balance skills. Elite athletes may develop neural adaptations related to tasks involving the spine and vertebral levels, effectively reducing the excitability of spinal and stretch reflexes during postural tasks [6,7]. Such adaptations minimize instability during movement and improve overall balance proficiency. Research consistently indicates that elite athletes outperform lower-level athletes in balance tasks [8,9]. For example, elite rifle shooters possess superior bipedal static balance than both national and novice-level shooters[10]. Similarly, national-level soccer players outperform regional-level players in unipedal and bipedal static balance, as well as unipedal dynamic balance. These distinctions are critical, as balance ability directly impacts the execution of specific motor skills and ultimately determines success in competitive environments.

Previous methods used to assess balance control in athletes have several limitations. Traditional approaches predominantly depend on static posturography [11], which primarily measures the body’s ability to maintain balance in a stationary position. While this method has its merits, it does not fully capture the more dynamic aspects of balance control required in many sports. For example, static posturography tracks the displacement range of the center of pressure (COP) in both the anteroposterior (AP) and mediolateral (ML) directions, providing only a partial view of an athlete’s overall balance capabilities [3]. Traditional time-domain features, like mean and root mean square (RMS), have been commonly employed to assess balance control [12]. In recent years, various signal processing methods, such as entropy analysis and detrended fluctuation analysis, have been introduced to more effectively capture these features [13,14]. However, despite these advancements, traditional time-domain classification methods remain significantly challenged [15]. They often fail to account for the complexity and dynamism inherent in athletic balance control. Furthermore, their ability to describe the non-linear and non-stationary characteristics of balance signals is limited, yet these features are essential for accurate assessment and classification in sports contexts.

In the analysis of postural stability, advanced methods such as the Hilbert-Huang Transform (HHT) offer significant insights. HHT efficiently characterizes the quality of balance control by analyzing time series data [16,17]. Studies have demonstrated that balance deteriorates after exposure to vibration, as reflected in the increased values of Intrinsic Mode Functions (IMFs) for both anteroposterior (AP) and mediolateral (ML) displacements. Specifically, the increase in IMF values across different IMFs indicates balance degradation following vibration. While traditional analytic signals may lack clear geometric patterns due to the influence of multiple control strategies, the application of HHT enables the identification of distinct circular geometries for each IMF, providing a more refined understanding of postural control [18].

This study introduces an innovative approach called the Composite Multiscale Complexity Index (CMCI) to enhance the assessment and classification of athletes’ balance performance. 1) Our approach integrates both static and dynamic balance parameters while incorporating Hilbert-Huang Transform (HHT) features to capture the non-linear and non-stationary nature of balance signals, providing a more comprehensive assessment of balance control. 2) We discussed the effectiveness of various machine learning methods in developing models to evaluate and predict athletes’ balance performance, particularly in dynamic sports such as freestyle aerial skiing and among groups with subtle balance differences.

## 2 Materials and methods

### 2.1 Subjects and procedures

This study involved two groups of participants: 13 elite athletes who competed in the Beijing Winter Olympics or World Championships (age: 22.50 ± 4.5 years; height: 174.68 ± 2.2 cm; weight: 70.46 ± 4.2 kg), 12 expert athletes who participated in national competitions (age: 20.00 ± 2.8 years; height: 174.87 ± 4.4 cm; weight: 69.22 ± 6.7 kg). All participants were in good physical condition and free from sports injuries or other diseases in the six months prior to testing, ensuring stable performance during the tests. The athletes were not fatigued during the tests, which were scheduled on their rest days. The recruitment period for this study started on 15/03/2020 and ended on 30/05/2022. This study was approved by the ethics committee2018(09).

A portable balance device (Humac Balance, USA), measuring 65 cm × 40 cm with a sampling frequency of 100 Hz, was used as the stable support surface for participants. To introduce a destabilizing factor to the athletes’ proprioception, a foam pad measuring 65 cm × 40 cm and 50 mm in thickness was employed. The athletes’ Center of Pressure (COP) on both stable and unstable support surfaces was recorded using the balance device.

Participants were instructed to remove their shoes and socks before standing, aiming to "maintain body trunk stability and minimize movement as much as possible." A quiet environment was maintained throughout the test to ensure optimal testing conditions. Following a standardized protocol, participants were instructed to place their hands on their hips, aligning the bottom of their second metatarsal bone with the marker on the balance device.

**Fig 1:**
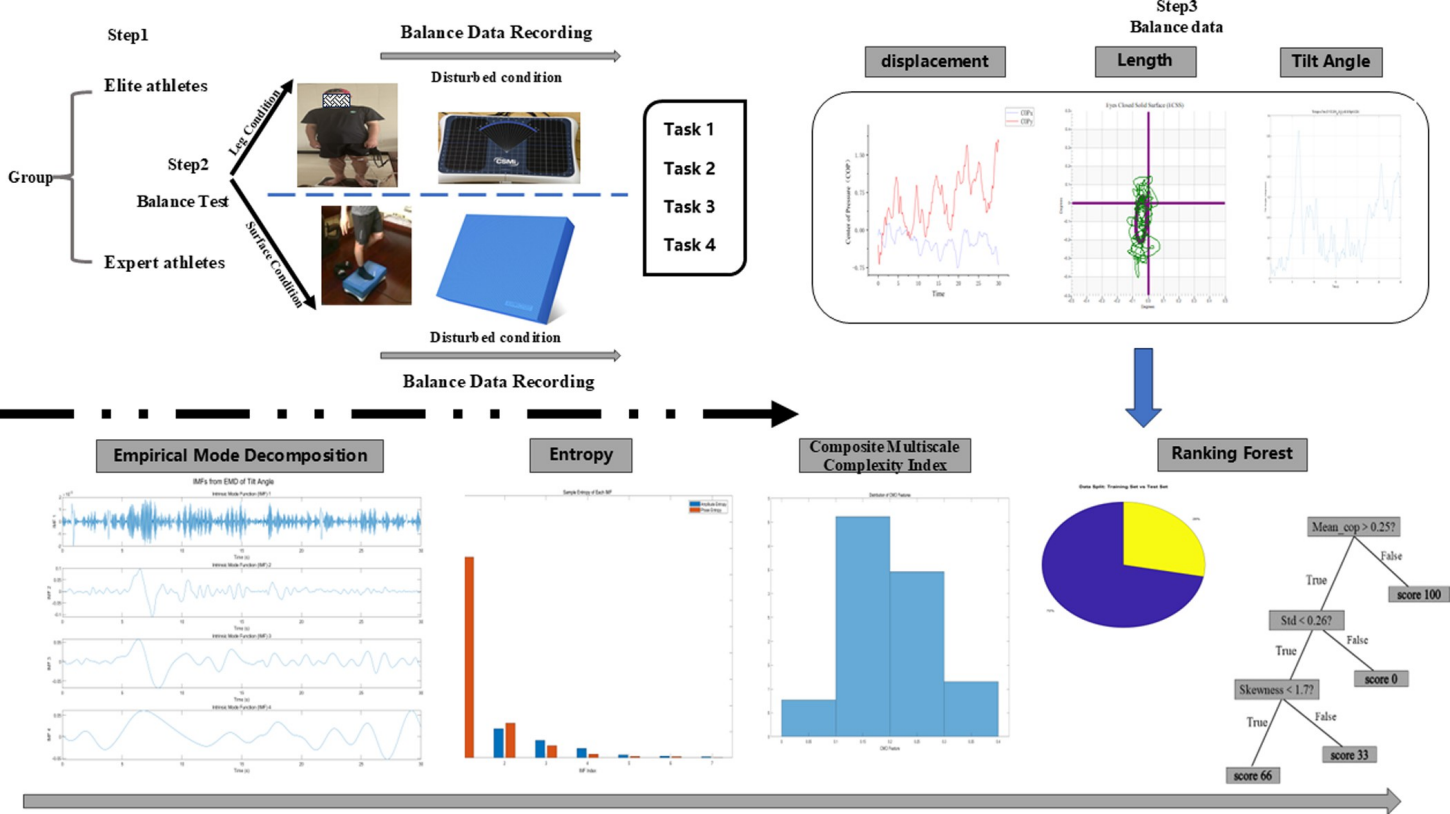
Experimental set-up and data analysis. (a) data acquisition process. In Step 1, We selected 13 elite freestyle skiing aerials athletes and 12 expert freestyle skiing aerials athletes. Step 2 involves conducting two experiments on all participants: First, 30-second balance recording test during various support leg conditions, and second, a 30-second test under varying surface conditions. Throughout this process, detailed Balance data for all participants are meticulously recorded. In Step 3, we primarily record the athletes’ displacement in the X and Y directions of cop the cop length, and the tilt angle throughout the entire testing process. (b) The data analysis process includes performing Signal Decomposition, Hilbert Transform, Sample Entropy, Total Complexity, Composite Multiscale Complexity Index, and machine learning on all collected balance data.

Participants were instructed to focus their gaze on a white circle with a diameter of 5 cm, positioned at eye level on a wall 3 meters away, while avoiding any other movements. Throughout the experiment, participants maintained forward gaze, with a slight bend in the hip and knee joints. The static standing posture was tested under four different conditions: T1: Both legs standing on a firm surface; T2: Both legs standing on a foam surface; T3: Both legs standing on a firm surface with eyes closed; T4: Both legs standing on a foam surface with eyes closed. Each standing condition lasted for 30 seconds, with a 30-second seated rest period between conditions (Fig 1).

### 2.2 Data description and processing

#### 2.2.1 Balance data

Center of pressure (COP) coordinates were analyzed in MATLAB 2022a (Mathworks, Natick, MA, USA) to calculate displacement, Length, related parameters in anterior-posterior, mediolateral, and Tilt angle (Table 1).

**Table 1:** Balance parameters description.

Parameters	Description
Patient COP (X, Y)	The patient’s Center of Pressure in inches where (0,0) is the patient’s center of balance (Inches).
COP Len	The length in inches of the patient’s COP (Inches).
Tilt Angle	The patient’s Tilt Angle in Degrees (degrees).

In traditional balance control assessments, static postural methods are typically employed to evaluate the displacement of the Center of Pressure (CoP). However, these methods often fail to account for the dynamic aspects of balance control during movement. To address this limitation, we introduced the tilt angle as a novel parameter, derived from the Humac balance system, to provide a more comprehensive evaluation of athletes’ balance abilities [19].


$$COPLen=\sqrt{COP_{x}^{2}+COP_{y}^{2}}$$

$$Tilt\;Angle=\cos^{-1}\left(\frac{COPLen}{Patient_{height}\times 0.55}\right)$$

To evaluate postural stability, we calculate the Tilt Angle by analyzing the displacement of the COP from its equilibrium position. The COP is derived using the coordinates *COP*_*x*_ and *COP*_*y*_, which represent the body’s pressure center on the horizontal plane. The resultant displacement, denoted as *COP*_*Len*_. The Tilt Angle is then derived by using 55% of the patient’s height to approximate the location of the Center of Mass (COM), providing a quantitative measure of the body’s inclination from the vertical axis. This angle is a critical indicator of the patient’s balance and stability across various postural tasks.

#### 2.2.2 Composite Multiscale Complexity Index (CMCI)

The input signal for the Complex Modulation and Coupling Index (CMCI) analysis consists of Center of Pressure (CoP) data recorded during static balance tests. Specifically, the CoP data was collected from participants standing on both stable and unstable surfaces, including a firm surface and foam, under various conditions (with eyes open and closed).

(1) Signal Decomposition:

The signal is decomposed into a finite number of Intrinsic Mode Functions (IMFs) using Empirical Mode Decomposition (EMD). This decomposition occurs through an iterative sifting process, where local maxima and minima are identified to generate upper and lower envelopes. The mean of these envelopes is then subtracted from the original signal, isolating each IMF. The sifting continues until a predefined stopping criterion is met, which is typically based on the standard deviation between successive siftings. To ensure that the IMFs reflect the most relevant oscillatory modes of the signal, mode truncation is applied by discarding IMFs that exhibit negligible energy or represent irrelevant frequency components. This truncation process helps eliminate noise and ensures that the retained IMFs capture meaningful signal characteristics [20].


$$S(t)=\sum_{i=1}^{n}IMF_{i}(t)$$

The signal *S*(*t*) is decomposed into a finite number of Intrinsic Mode Functions (IMFs) using EMD. Each IMF represents a simple oscillatory mode embedded in the signal [21].

(2) Hilbert Transform:

$$H\left(IMF_{i}(t)\right)=a_{i}(t)e^{j\varphi_{i}(t)}$$

The Hilbert transform is applied to each IMF *IMF*_i_(*t*) to obtain its analytical signal. The analytical signal is represented as a complex function where *a*_i_(*t*) is the instantaneous amplitude and *φ*_i_(*t*) is the instantaneous phase of the *i-th* IMF.

(3) Sample Entropy:

Sample Entropy (SE) is a measure of signal complexity, reflecting the unpredictability or irregularity of time-series data. In this study, SE is calculated for both the instantaneous amplitude and phase of each Intrinsic Mode Function (IMF) obtained through the Empirical Mode Decomposition of the COP signal. The SE calculation involves defining two key parameters: the embedding dimension *r*. Where *m* = 2 represents the length of subsequences being compared, and *r* = 0.2 × std represents the similarity criterion, based on 20% of the standard deviation of the signal[22]. For each IMF, SE measures the likelihood that similar subsequences remain similar as the sequence extends by one additional point. It is computed using the following formula:

$$SE\left(a_{i}(t)\right),SE\left(\varphi_{i}(t)\right)$$

SE is calculated for both the instantaneous amplitude *a*_i_(*t*) and the instantaneous phase *φ*_i_(*t*) of each IMF. SE is a measure of uncertainty or complexity within a signal.

(4) Total Complexity:

The total complexity of each IMF is calculated by summing the weighted entropies of both the instantaneous amplitude and instantaneous phase. To ensure that both components contribute meaningfully to the overall complexity measure, appropriate weights must be assigned to the entropies of amplitude and phase. In this analysis, the weights for the amplitude and phase entropies are assumed to be equal as the goal is to assess the overall complexity of the signal without prioritizing one component over the other.


$$Total\;Complexity_{i}=\sum_{i=1}^{n}\omega_{i}\left(SE\left(a_{i}(t)\right)\right)+SE\left(\varphi_{i}(t)\right)$$

The total complexity for each IMF is computed by summing the weighted entropies of both the amplitude and phase. *ω*_*i*_ represents the weight associated with each entropy term.

(5) Composite Multiscale Complexity Index (CMCI):

$$CMCI=\frac{1}{m}\sum_{i=1}^{m}Total\;Complexity_{i}$$

The Composite Multiscale Complexity Index (CMCI) is determined by averaging the total complexities of all IMF components. This index provides a comprehensive measure of the signal’s overall complexity across multiple scales.

### 2.3 Classification techniques

We employed two distinct feature extraction techniques for classification: original time-domain features and those derived from the Composite Multiscale Complexity Index (CMCI). The original time-domain features included the mean, standard deviation, skewness, and kurtosis, which were extracted from balance signals across four tasks. CMCI features were derived by applying the CMCI process to individual variables, as described in section 2.2.2.

To analyze the performance in evaluating our predictive approach, we employed a standard train-test methodology. The dataset, comprising all collected balance parameters labeled across two groups—elite athletes and regular athletes—was randomly split into a training set (70% of the data) and a test set (30%). We ensured that the proportion of elite and regular athletes was balanced in both sets to maintain consistency.

Classification is utilized to ascertain the category (group or class) to which a new observation or instance belongs, based on a training dataset containing instances with known category memberships. Classification techniques typically encompass three approaches: statistical methods, machine learning techniques, and neural network methods [23–25]. Considering these approaches, we employed four common classifiers to predict and distinguish balance ability similarities among various athletes.

Ranking Forest: We utilized the Ranking Forest algorithm for its robustness in handling high-dimensional data and its capacity to capture complex relationships among features. This method utilizes bagging to construct and aggregate 50 decision trees, each built on random subsets of the training data and features. Ranking Forest is particularly effective in providing stable predictions and reducing the risk of overfitting, thus making it particularly suitable for our balance data, which can be highly variable [26].

Naive Bayes: Naive Bayes was chosen due to its simplicity and efficiency, particularly when dealing with high-dimensional feature spaces. Although it assumes that features are conditionally independent—an assumption that may not always hold true in balance data—its probabilistic nature establishes it as a strong baseline classifier. Naive Bayes is particularly useful for understanding the contribution of individual features to the classification task, offering insights into which balance parameters are most predictive [27].

Logistic Regression: Logistic Regression is a widely used algorithm for binary classification tasks, perfectly aligning with our need to classify athletes into two groups. It models the probability that a given input belongs to a particular class, making it valuable for interpreting the importance of different balance features. Logistic Regression is particularly useful for understanding linear relationships between the features and the outcome, rendering it a suitable choice for initial analysis and interpretation of the balance data [28].

Support Vector Machines (SVM): SVM is recognized for its effectiveness in high-dimensional spaces and its capacity to handle non-linear relationships through kernel functions. Given the complex and non-linear nature of balance data, this makes SVM well-suited to identify subtle distinctions between elite and regular athletes. By maximizing the margin between different classes, SVM establishes a robust classification framework, particularly in cases where class balance is critical [29].

#### 2.3.1 Evaluation

Following the described methodology, we utilized both CMCI and original time-domain features for training and evaluation. Labels were predicted for the test set, and ROC curves were plotted to calculate the AUC for each feature set. Additionally, to ensure a comprehensive performance evaluation, we performed 10-fold cross-validation to compute the average AUC, sensitivity (SEN), and specificity (SPC) across all classifiers.

For statistical analysis, the average and standard deviation of each descriptor were reported for both populations. Normality was assessed using the Kolmogorov-Smirnov test. If normally distributed, pairwise comparisons were conducted using the independent samples t-test; otherwise, the Mann-Whitney U test was applied. Significance was determined at *P*<0.05. AUC for each descriptor was calculated using a one-dimensional Support Vector Machine with a soft margin.

## 3 Results

### 3.1 Subject’s characteristics

Table 2 provides an overview of the balance performance characteristics of the participants in this study. The analysis revealed no statistically significant differences between elite and expert athletes in the balance parameters COPLen, COPX, COPY, and Tilt angle across tasks T1, T2, and T3. However, a significant difference was observed in the COPY parameter during task T4 (*P*<0.05). These findings suggest that traditional balance measurement parameters may not sufficiently differentiate between elite and expert athletes. Given the essential role of balance in athletic performance, distinguishing between these two groups is crucial. This raises the question of whether these parameters are adequate for accurately classifying and distinguishing the similar balance abilities of elite and expert athletes.

**Table 2:** Balance performance of elite and expert athletes.

Condition	Parameters	Elite athlete	Expert athlete
Firm surface	COPX	0.04(0.2)	0.11(0.2)
	CMCI-COPX	0.26(0.08)	0.23(0.07)
	COPY	0.01(0.4)	-0.01(0.5)
	CMCI-COPY	0.23(0.06)	0.25(0.09)
	COPLen	0.28(0.3)	0.43(0.3)
	CMCI-COPLen	0.26(0.06)	0.22(0.08)
	Tilt angle	0.21(0.1)	0.28(0.2)
	CMCI-Tilt angle	0.24(0.06)	0.25(0.08)
Foam surface	COPX	0.05(0.9)	-0.03(0.7)
	CMCI-COPX	0.24(0.1)	0.24(0.09)
	COPY	0.11(0.4)	-0.05(0.5)
	CMCI-COPY	0.22(0.08)	0.20(0.07)
	COPLen	0.53(0.7)	0.65(0.2)
	CMCI-COPLen	0.16(0.06)*	0.22(0.07)
	Tilt angle	0.31(0.1)	0.40(0.1)
	CMCI-Tilt angle	0.18(0.08)	0.22(0.07)
Firm surface with eyes closed	COPX	0.4(0.04)	0.05(0.2)
	CMCI-COPX	0.26(0.08)	0.24(0.1)
	COPY	0.11(0.1)	-0.02(0.3)
	CMCI-COPY	0.20(0.08)*	0.27(0.09)
	COPLen	0.69(0.1)	0.28(0.2)
	CMCI-COLen	0.20(0.08)	0.25(0.09)
	Tilt angle	0.31(0.1)	0.40(0.1)
	CMCI-Tilt angle	0.25(0.08)	0.24(0.08)
Foam surface with eyes closed	COPX	-0.04(0.2)	0.08(0.2)
	CMCI-COPX	0.24(0.06)	0.27(0.08)
	COPY	0.47(0.3)*	0.04(0.4)
	CMCI-COPY	0.25(0.08)	0.24(0.07)
	COPLen	0.5(0.2)	0.55(0.2)
	CMCI-COPLen	0.23(0.1)	0.24(0.67)
	Tilt angle	0.47(0.1)	0.55(0.4)
	CMCI-Tilt angle	0.20(0.08)	0.25(0.08)

All values are presented as means ± standard deviations. *P < 0.05 indicates a significant difference between elite athletes and the expert athlete group. Normality was assessed using the Kolmogorov-Smirnov test. If normally distributed, pairwise comparisons were conducted using the independent samples t-test; otherwise, the Mann-Whitney U test was applied.

### 3.2 Performance of computational models

**Fig 2:**
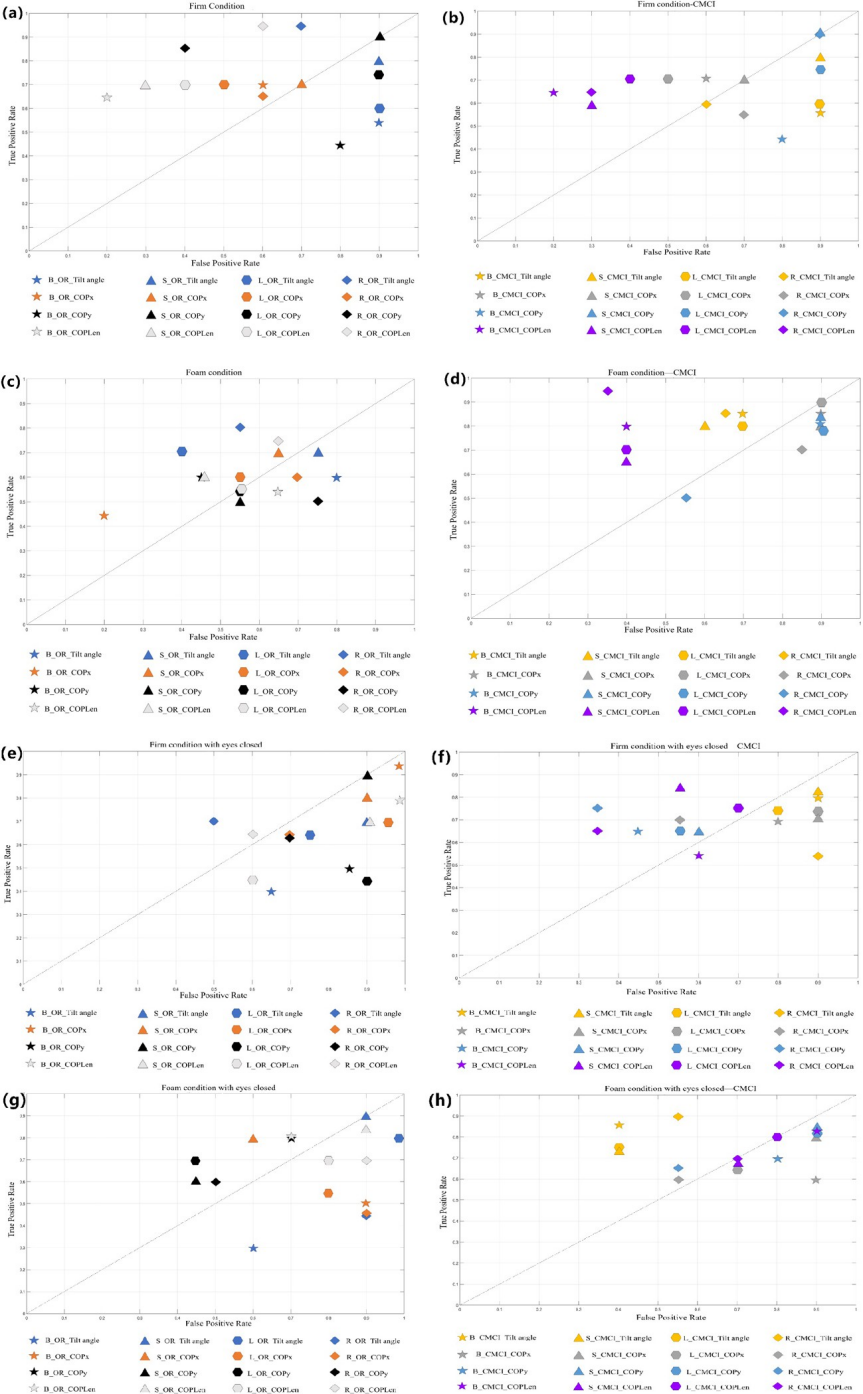
Model performance in ROC space across different algorithms and task conditions. Performance of models in ROC space for datasets under all conditions (firm surface with eyes open and eyes closed, foam surface with eyes open and eyes closed), using all data processing methods (original time domain and CMCI method), and all classification methods (Bayesian, Ranking Tree, Logistic Regression, Support Vector Machine). (a, c, e, g) represent the performance of all classifiers using the original time domain processing method, while (b, d, f, h) represent the performance of all classifiers using the CMCI processing method. Each model’s name follows the following naming rules: The first uppercase letter represents the name of the classifier (e.g., SVM: S, Logistic Regression: L, Bayesian: B, Ranking Tree: R); the second word indicates the data processing method, where OR represents the original time domain processing method, and CMCI represents data processing using the CMCI method; the last part of the name indicates the variable names used.

The results of the models are presented in two distinct sections. First, we analyzed the classification performance of various classifiers on datasets processed in two ways, illustrating the true positive rate and false positive rate for each developed model within their receiver operating characteristic (ROC) space (Fig 2). Balancing the dataset using CMCI techniques typically results in improved classification performance. The cluster of CMCI-processed data resides in the upper left quadrant of the ROC space (above the reference line), indicating optimal classifier performance. In contrast, the performance of datasets utilizing only original data approaches or falls below the reference line.

Subsequently, we conducted a comprehensive analysis of the classifiers, including their performance under various test conditions and data processing methods, demonstrating enhanced performance in ROC space (Fig 2). Specifically, models constructed using the Ranking Forest classifier in conjunction with CMCI parameters typically exhibited the highest performance, achieving a sensitivity of 0.95 and a specificity of 0.35. Subsequently, the Naive Bayes classifier demonstrated satisfactory performance under the eyes-closed condition, achieving a sensitivity of 0.85 and a specificity of 0.40. However, the impact of integrating CMCI with various classifiers is contingent upon the specific balance tasks and parameters, which will be elaborated upon in detail in Section 3.3.

### 3.3 Impact of task difficulty on classification accuracy

**Fig 3:**
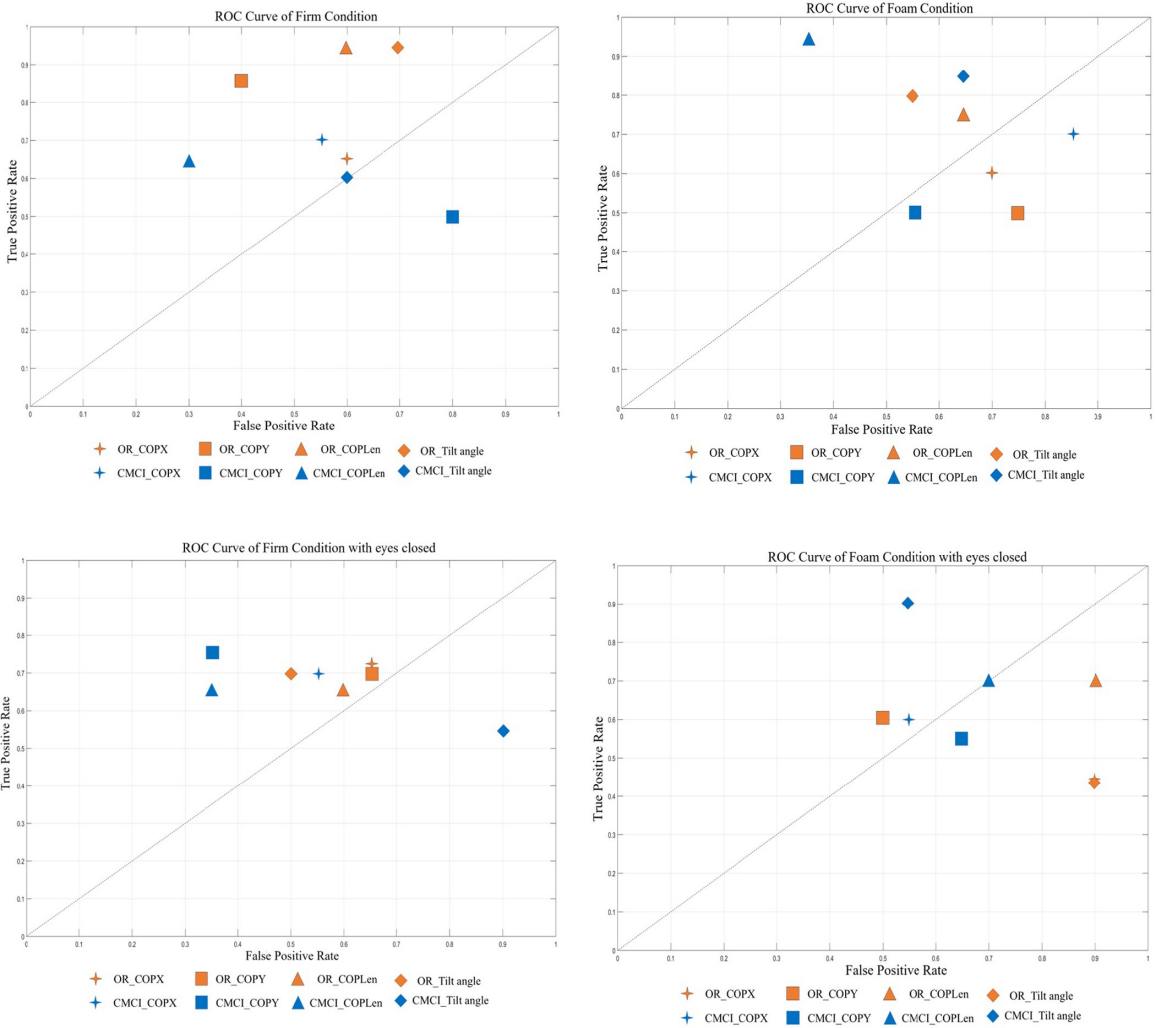
Ranking forest model performance in ROC space for all balance features. By applying the Ranking Forest machine learning algorithm to the dataset under all conditions (eyes-open firm surface, eyes-closed firm surface, eyes-open foam surface, and eyes-closed foam surface), we evaluated the ROC space of the models for all balance features (both original parameters and those processed using the CMCI method). The models developed using the datasets processed with the CMCI technique exhibited superior performance. Each model’s name follows the naming convention described below: The first uppercase letters indicate the set of variables used, with OR = original features, CMCI = variables processed with the CMCI method, and (a, b, c, d) representing different balance tasks(T1-T4).

Based on the results presented in Section 3.2, we evaluated the classification performance of the CMCI in conjunction with the Ranking Forest method on data from four distinct balance tasks in ROC space (Fig 3), assessing how varying tasks influenced classification accuracy. Specifically, for Task T1, neither the time-domain feature extraction nor the CMCI-processed data yielded satisfactory classification results with the Ranking Forest method. Notably, the ROC analysis of time-domain features indicated performance akin to a random classifier, with AUC values fluctuating between 0.49 and 0.54. Nevertheless, as task difficulty increased, the classification performance of the CMCI-processed COPLen variable exhibited the highest accuracy when utilizing the Ranking Forest method (sensitivity = 0.95, specificity = 0.35). As task difficulty further escalated, even under the eyes-closed condition, CMCI-COPLen persisted in demonstrating robust classification performance (sensitivity = 0.75, specificity = 0.35). Ultimately, in Task 4, the most challenging condition, none of the raw data features yielded accurate classification, while only the CMCI-processed tilt angle distinguished itself (sensitivity = 0.9, specificity = 0.55).

### 3.4 Construct validity

The CMCI processing method selected features associated with COP displacement and tilt angle across four tasks (firm and foam surfaces with eyes open and closed). CMCI-processed features surpassed the original time-domain features (COPX, COPY, COPLen, and tilt angle) in classification accuracy, particularly in high-difficulty tasks, such as balancing on a foam surface, which disrupts proprioceptive feedback. This improvement aligns with theoretical expectations that more challenging balance tasks should yield more significant results in assessing postural stability. Conversely, the original features demonstrated poor performance under difficult conditions, indicating limitations in distinguishing between various balance states.

The superior performance of CMCI-processed features in high-difficulty tasks supports the construct validity of these features, whereas the inferior performance of the original features raises questions about their construct validity. This discrepancy highlights the critical role of effective feature selection and processing in accurately capturing the construct of postural balance.

## 4 Discussion

It is well known that the Center of Pressure (CoP) platform has been extensively utilized by clinical researchers to further assess postural control in various populations [30]. Feature selection is informed by prior studies highlighting the significance of parameters, such as the range of motion in static balance tasks, including the medial-lateral and anterior-posterior ranges, and the mean distance of the CoP trajectory center [31]. More advanced local indicators, such as the distance between peaks in the sway density curve [32], may also enhance the prediction of balance capabilities through CoP trajectories. However, these methods encounter challenges in distinguishing subtle balance differences, particularly among athletes and injured populations. This difficulty arises because, although these features can capture certain aspects of balance control, they often fail to effectively reveal minor balance variations. Consequently, more precise analytical methods are necessary to improve sensitivity to balance differences. This demand for advanced analysis is substantiated by theoretical frameworks emphasizing the importance of refining measurement techniques to detect subtle changes in postural control, particularly in specialized populations.

### 4.1 Balance classification performance for specific tasks

In our study of elite and expert athletes, the differences in balance ability between the two groups are minimal. We found it nearly impossible to classify them accurately using any features extracted from the time domain. We detected only slight differences between the two groups in the anterior-posterior direction during Task T4, which increases the demands for classification methods and data processing. Extensive prior research on balance classification has demonstrated that traditional balance features are inadequate for achieving accurate classification results [33,34]. Furthermore, we observed that classifying balance in simple tasks presents significant challenges. This difficulty may stem from the lack of external disturbances during simple tasks, resulting in a more stable and regular balance, which makes it challenging to accurately distinguish between balance abilities.

However, as the difficulty of the balance tasks increased, we observed that various tasks and external factors had a significant impact on classification accuracy. For instance, the introduction of proprioceptive interference to the athletes enhanced the classification accuracy of combinations of time-domain features and tilt angle parameters. This finding suggests that proprioceptive interference is more effective than visual interference in detecting subtle balance differences among individuals. Moreover, the classifier’s performance was significantly improved when employing the innovative CMCI approach. This implies that traditional feature analysis alone is inadequate for distinguishing subtle differences in balance ability. The CMCI method appears more suitable for differentiating between populations with minimal balance differences, particularly among skill-based athletes, where such differences are minimal. Furthermore, the results of traditional statistical tests must be interpreted with caution. Tests such as rank-sum tests indicate significant differences between two distributions; however, statistical differences do not necessarily yield effective solutions for classification problems. Additional information is required to classify new participants as elite or expert athletes.

Finally, does increasing the difficulty of balance tasks always enhance classifier accuracy? The answer is no; increasing task difficulty does not always lead to improved classifier accuracy. Our balance tests encompass simple tasks (T1, T2) and more challenging tasks (T3, T4), yet we did not observe the highest classification accuracy in T3 and T4. Traditionally, it is believed that more challenging balance tasks improve classifier performance by revealing greater postural sway and irregular movements. However, our study revealed that when both visual and proprioceptive interference were present, the classification accuracy between elite and expert athletes was suboptimal. This may stem from the increased difficulty, which causes athletes to struggle to maintain stable balance, resulting in greater randomness and uncontrollability [35,36]. Consequently, the relationship between balance test difficulty and classifier accuracy is intricate, particularly in detecting subtle differences in balance ability.

### 4.2 Distinguishing balance ability through the tilt angle

Prior studies have indicated that combinations of multiple features can partially reflect postural instability [37,38]. Although individual features (such as medial-lateral sway) are useful, they often fail to accurately differentiate between various populations, consistent with our findings. We also examined the impact of single features on classifier performance. Common indicators traditionally employed to evaluate balance ability include anterior-posterior and medial-lateral center of pressure displacement, center of pressure movement path, and center of pressure velocity. In contrast, we propose the utilization of the body’s tilt angle as a novel parameter for assessing balance ability. Our findings indicate that the tilt angle parameter extracted from time-domain features consistently improved the performance of various classifiers across nearly all standing tasks. Consequently, future research aimed at differentiating and revealing balance differences among diverse populations should consider the tilt angle as a key metric, particularly across various standing tasks. The tilt angle may represent one of the more appropriate indicators for balance assessment.

Theoretically, this approach aligns with the notion that balance encompasses both static and dynamic aspects of postural control. Traditional features typically emphasize the displacement and movement of the center of pressure, primarily capturing dynamic changes while potentially overlooking static postural adjustments. Tilt angle, as a measure of the body’s alignment relative to the vertical, provides a direct assessment of static balance stability, complementing dynamic measures. This theoretical foundation supports the notion that incorporating tilt angle could yield a more comprehensive evaluation of balance, particularly in distinguishing populations with subtle differences in balance ability.

### 4.3 The power of non-linear models and CMCI in balance assessment

Previous research has also incorporated multiple indicators in their instability assessment models [39]. Reports indicate that employing machine learning methods to analyze multidimensional features can significantly enhance classification performance [40]. For example, earlier studies have employed ranking forests to differentiate between fallers and non-fallers based on static postural graphs, achieving an AUC of 0.75 [41]. These studies suggested that the algorithms relied on a straightforward linear combination of features. However, the nature of feature interrelationships can be highly complex, and linear models may not sufficiently capture these relationships. The inherently deep non-linear nature of ranking forest algorithms enables them to model more complex and generalized patterns [42]. In our study, we employed the CMCI method to extract features from the raw data and subsequently applied ranking forests for classification analysis. We discovered that the integration of CMCI with ranking forests significantly enhanced the classifier’s performance. Non-linear signal decomposition methods can extract features that reveal subtle variations in balance control, which are often challenging to detect using linear methods [43,44]. For instance, adaptive responses of the balance system to perturbations induce neuromuscular reactions across various frequency bands and time scales, which can be captured through non-linear feature extraction [45]. When these features are integrated into advanced classification models, they reveal deeper patterns related to balance control, thereby enhancing classification accuracy.

This enhancement can be attributed to the capacity of non-linear models to capture complex dependencies between features that linear models may overlook. CMCI, as a feature extraction method, identifies subtle variations in balance data that may not be apparent through traditional features. When combined with the non-linear capabilities of ranking forests, this approach enables a more nuanced understanding of balance instability, resulting in improved classification performance. These findings highlight the potential of integrating advanced feature extraction and non-linear modeling techniques to improve the accuracy and effectiveness of balance assessments.

### 4.4 Limitations

We assessed balance ability in elite and expert athletes across only four types of balance tasks. The study focused exclusively on two groups exhibiting minimal differences in balance ability. Future research should encompass a broader range of populations to enhance generalizability. Additionally, the selected indicators were based on commonly used metrics from prior literature and were not subjected to further screening. Future studies should incorporate a wider array of balance parameters to validate the classifier’s results and evaluate the feature recognition capabilities of CMCI for additional indicators.

## 5 Conclusion

This study presents a novel method for classifying balance ability differences between elite and expert athletes. The approach is based on the Ranking Forest algorithm, which combines the robustness of classification performance with the advantage of requiring only simple balance measurements. In our model, the integration of CMCI features with Ranking Forests demonstrated a significant improvement in classifier performance compared to traditional methods. The use of CMCI facilitated a more detailed extraction of balance-related features, enabling us to identify differences between athletes with minimal balance variability. Additionally, the results suggest that classification accuracy may be influenced by specific balance tasks. Future research should explore applying this method to a broader range of populations and balance tasks to further validate and refine its effectiveness.
